# Clinical and immunological characteristics of tegumentary leishmaniasis cases in Bolivia

**DOI:** 10.1371/journal.pntd.0009223

**Published:** 2021-03-05

**Authors:** Cristina Ballart, Mary Cruz Torrico, Gisela Vidal, Faustino Torrico, Daniel Lozano, Montserrat Gállego, Lilian Pinto, Ernesto Rojas, Ruth Aguilar, Carlota Dobaño, Sonia Ares-Gomez, Albert Picado

**Affiliations:** 1 ISGlobal, Hospital Clínic—Universitat de Barcelona, Barcelona, Spain; 2 Secció de Parasitologia, Departament de Biologia, Sanitat i Medi Ambient, Facultat de Farmàcia i Ciències de l’Alimentació, Universitat de Barcelona, Barcelona, Spain; 3 Fundación CEADES Salud y Medio Ambiente, Cochabamba, Bolivia; 4 Facultad de Medicina, Universidad Mayor de San Simón (U.M.S.S.), Cochabamba, Bolivia; 5 Foundation for Innovative New Diagnostics (FIND), Geneva, Switzerland; University of Notre Dame, UNITED STATES

## Abstract

**Background:**

Tegumentary leishmaniasis (TL) is a parasitic disease that can present a cutaneous or mucocutaneous clinical form (CL and MCL, respectively). The disease is caused by different *Leishmania* species and transmitted by phlebotomine sand flies. Bolivia has one of the highest incidences of the disease in South America and the diagnosis is done by parasitological techniques. Our aim was to describe the clinical and immunological characteristics of CL and MCL patients attending the leishmaniasis reference center in Cochabamba, Bolivia, in order to gain updated clinical and epidemiological information, to evaluate the diagnostic methods used and to identify biomarkers related to clinical disease and its evolution.

**Methodology/Principal findings:**

The study was conducted from September 2014 to November 2015 and 135 patients with lesions compatible with CL or MCL were included. Epidemiological and clinical data were collected using a semi-structured questionnaire. Two parasitological diagnostic methods were used: Giemsa-stained smears and culture of lesion aspirates. Blood samples obtained from participants were used to measure the concentrations of different cytokines. 59.2% (80/135) were leishmaniasis confirmed cases (CL: 71.3%; MCL: 28.7%). Sixty percent of the confirmed cases were positive by smears and 90.6% were positive by culture. 53.8% were primo-infections. Eotaxin and monokine induced by IFN-γ presented higher serum concentrations in the MCL clinical presentation compared to CL cases and no-cases. None of the cytokines presented different concentrations between primo-infections and secondary infections due to treatment failure.

**Conclusions/Significance:**

In Bolivia, parasitological diagnosis remains the reference standard in diagnosis of leishmaniasis because of its high specificity, whereas the sensitivity varies over a wide range leading to loss of cases. Until more accurate tools are implemented, all patients should be tested by both smears and culture of lesion aspirates to minimize the risk of false negatives. Our results showed higher concentrations of several cytokines in MCL compared to CL, but no differences were observed between CL and no-cases. In addition, none of the cytokines differed between primary and secondary infections. These results highlight the need of further research to identify biomarkers of susceptibility and disease progression, in addition to looking at the local cellular immune responses in the lesions.

## Author summary

Tegumentary leishmaniasis is a parasitic disease caused by different *Leishmania* species and transmitted by phlebotomine sand flies. The disease is distributed worldwide and it can present a cutaneous or mucocutaneous clinical form. The clinical outcomes of the disease depend on factors inherent to the parasite, the vector and the host. Bolivia has one of the highest incidences of the disease in South America, where the diagnosis is mainly done by parasitological techniques. The present study was conducted from September 2014 to November 2015 with 135 patients attending the leishmaniasis reference centre in Cochabamba (Bolivia). We aimed to describe the clinical, epidemiological and immunological characteristics of leishmaniasis patients to gain knowledge on tegumentary leishmaniasis and evaluate the suitability of the diagnostic methods used. In South America, Giemsa-stained smears and the culture of lesion aspirates remain the reference standards in diagnosis because of their high specificity, however the sensitivity varies over a wide range resulting in some loss of case detection. Until more accurate tools are implemented, all patients should be screened by both tests to minimize the risk of false negatives. Additionally, for better disease management, identification of biomarkers of susceptibility and disease progression would be desirable.

## Introduction

Tegumentary leishmaniasis (TL) has two different clinical presentations: ulcerative skin lesions (cutaneous leishmaniasis [CL]) or a destructive mucosal inflammation (mucocutaneous leishmaniasis [MCL]) that affects oral-nasal-pharyngeal cavities. MCL usually appears after a CL episode but both clinical forms can present together [1]. The clinical outcomes of the disease depend on factors inherent to the parasite, the vector, and the host. CL patients should be diagnosed and treated even if CL lesions may self-resolve in up to 70–80% of cases, as they can cause a high degree of morbidity, social stigmatization, and in some cases evolve to MCL [1].

Bolivia is one of the endemic countries with the highest incidence of CL and MCL in South America [1,2]. The *Leishmania (Viannia) braziliensis* species, transmitted by phlebotomine sand flies, is the predominant etiological agent causing CL in Bolivia (85% of cases) [1,2,3]. CL is endemic to 70% of Bolivia, with 54.7 cases per 100,000 individuals in 2018 [1,4,5,6].

In Bolivia, diagnosis of leishmaniasis is done by Parasitological Direct Exam (PDE) and in a few cases, by parasite culture method (CM). Patients cover the cost of both diagnostics. TL treatment is free of charge but it is only provided when the case has been confirmed. In Bolivia, first-line TL therapy is meglumine antimoniate (Glucantime), administered by intravenous or intramuscular injection, depending on the clinical form of the disease (CL and MCL, respectively), at a dosage of 20 mg SbV/kg/day over a period of 20 to 30 days. Second-line treatment is amphotericin B deoxycholate (Fungizone) at a dosage of 0.5-1mg/kg/day administered intravenously for 30 to 45 days requiring a prolonged hospitalization [4,7,8]. However, therapy failures and variable efficacy of these drugs have been reported [1,7,9].

This study aimed to describe the clinical and immunological characteristics of CL and MCL patients attending the leishmaniasis reference center in Cochabamba. The study is relevant to have a realistic description of the population affected by TL in this low-income country area, and to identify potential problems associated with disease management, such as gaps in the diagnostic tools currently used. The study also assessed the usefulness of cytokines as biomarkers of clinical presentation and treatment failure, and highlights the need for further research on biomarkers of susceptibility and disease progression in leishmaniasis.

## Materials and methods

### Ethics statement

The study was approved by the Ethics Committees of the Hospital Clínic de Barcelona, Spain (HCB/2014/0582); CEADES Salud y Medio Ambiente and the Facultad de Medicina UMSS in Bolivia.

All suspected cases of leishmaniasis provided written informed consent (parents or guardians in case of patients were under 18 years old) before enrolling in the study. All leishmaniasis suspects attending LABIMED during the study period, including those not enrolled in the study, were diagnosed for free. CL and MCL cases were referred for treatment.

### Study population, data and sample collection

The study was conducted in patients attending the Institute of Biomedical Research, IIBISMED, and Laboratorios de Investigación Biomédica (LABIMED), from the Faculty of Medicine of the Universidad Mayor de San Simón (UMSS) in Cochabamba city (Bolivia). Suspects of leishmaniasis are referred to the IIBISMED from other health centres for diagnosis and treatment.

Patients with lesions compatibles with CL or MCL attending the LABIMED from September 2014 to November 2015 were invited to participate in the study. Subjects from both sexes and all ages were recruited. After obtaining written informed consent, data on the patients’ demographic, epidemiological and clinical characteristics (number, location and lesion type, time of evolution, previous drug treatment, duration, and outcome) were collected using a semi-structured questionnaire (S1 Fig). Smears and aspirates of active lesions were obtained to diagnose CL and MCL cases. More than one sample was collected per patient if multiple lesions were present. Blood samples were also obtained by venipuncture.

### Diagnostic methods

The diagnostic methods used were: i) microscopic examination of lesion smears obtained by scrape and stained with Giemsa for the visualization of amastigotes (PDE) and ii) culture of lesion aspirates from areas of apparent parasitic activity in TSTB (Torrico-Solano-Torrico-Bermúdez) biphasic medium (CM) [10]. Cultures were maintained for four weeks and considered either positive or negative if parasite growth was detected or not and considered contaminated if non-specific microorganisms were also present. PDE and CM were performed on samples from three different areas of the same lesion for all the patients who participated in the study.

### Definitions

Based on the clinical and epidemiological data and the results of the diagnostic tests, the following groups of patients were defined.

#### Clinical forms

Individuals who were positive by microscopy and/or culture were considered leishmaniasis-confirmed patients. Patients negative by microscopy and culture were considered no-cases. Patients presenting only cutaneous lesions in any area of the body were classified as CL, and those with mucosal lesions in mouth and/or nostrils, or concomitant cutaneous and mucosal lesions, were classified as MCL.

#### Primo and secondary infections

CL patients who had no previous history of leishmaniasis were defined as primo-infection (first episode). MCL patients with no history of CL or visible scars were considered to have primary lesions [11]. The rest were considered secondary infections (second episode).

#### Clinical cure and treatment failure

Clinical cure was defined as complete wound closure and re-epithelization without inflammation or filtration [12].

We used historical clinical data to identify patients who had a relapse due to treatment failure. CL and MCL patients who had previously received a complete anti-leishmanial treatment in the past but were not cured, regardless of its duration, were considered treatment failures. Those patients presented an absent or incomplete scarring of lesion(s) and/or a persistence of inflammation around the initial lesion, a clinical regression of a healed lesion or the presence of new mucosal lesion(s) [1,7,12,13]. For those cases with cutaneous lesions to be considered treatment failures, the previous treatment had to be directed to the same lesion(s) identified during our study.

### Cytokine, chemokine and growth factor multiplex bead array assay

The Cytokine Human Magnetic 30-Plex Panel from Life Technologies was used to measure the concentrations (pg/mL) of the following cytokines, chemokines and growth factors in sera: epidermal growth factor (EGF), fibroblast growth factor (FGF), granulocyte colony-stimulating factor (G-CSF), granulocyte-macrophage colony-stimulating factor (GM-CSF), hepatocyte growth factor (HGF), vascular endothelial growth factor (VEGF), tumor necrosis factor (TNF), interferon (IFN)-α, IFN-γ, interleukin (IL)-1RA, IL-1β, IL-2, IL-2R, IL-4, IL-5, IL-6, IL-7, IL-8, IL-10, IL-12(p40/p70), IL-13, IL-15, IL-17, IFN-γ induced protein (IP-10), monocyte chemoattractant protein (MCP-1), monokine induced by IFN-γ (MIG), macrophage inflammatory protein (MIP)-1α, MIP-1β, regulated on activation normal T cell expressed and secreted (RANTES) and eotaxin.

Twenty five μL of sera were tested by applying a modification to the manufacturer’s protocol by using half the volume of all reagents including the standards. This modification was previously tested and showed no difference in assay performance compared to the original protocol and has been used in prior studies [14,15]. Each plate included 16 serial dilutions (2-fold) of a standard sample with known concentrations of each analyte, two blanks and three positive controls of high, medium and low concentrations in duplicates, prepared from the standard for quality control purposes. Samples were acquired on a Luminex 100/200 instrument and analyzed with xPONENT software 3.1. Concentrations of analytes were obtained by interpolating the median fluorescent intensity (MFI) (after blank MFI subtraction) to a 5-parameter logistic regression curve calculated by xPONENT. Values below the lower limit of detection (mean of blanks + 2 standard deviations) were assigned half the expected concentration at the lower limit of quantification, and values above the upper limit of quantification were assigned twice the expected concentration at the upper limit of quantification.

### Statistical analysis

Clinical and epidemiological variables were all categorical and included: age, sex, occupation, clinical status, probable place of infection, episode of the disease, type and location of lesion, and questions related to treatment (previous treatments, drug used, dosage and resolution), among others. Differences in sex, age, number of episodes, characteristics of lesions, and characteristics of previous treatments between clinical forms, were assessed by Pearson’s Chi-square test at the significance level α = 0.05. The number of patients identified by both diagnostic techniques (PDE and CM) was estimated. The agreement between the diagnostic methods was evaluated using the Cohen’s kappa coefficient (K) calculated after excluding those cultures that were contaminated. Calculations were performed using the function epi.kappa from the epiR library in R version 3.5.2 [16].

Cytokine, chemokine and growth factor concentrations were tested for normality to inform the appropriate statistical analysis, showing a non-normal distribution. Differences in analyte concentrations between CL, MCL and no-cases, and between primo-infections and treatment failures (shown as box plots), were compared through the Kruskal-Wallis Chi-squared test adjusting for multiple comparisons by the Bonferroni correction. The effect of the clinical manifestation (CL or MCL) on analyte concentrations was assessed through univariable and multivariable linear regression models. Those analytes significant in the univariable models were included in a stepwise backward multiple regression selection at α = 0.05. The final selected model was adjusted by age. IBM SPSS Statistics version 24.0.0 (IBM Corp. Released 2016. IBM SPSS Statistics for Windows, Version 24.0.0 Armonk, NY: IBM Corp.) was used to conduct the statistical analyses related to the cytokine, chemokine, and growth factor concentrations.

## Results

From the 141 suspected leishmaniasis patients who reported to IIBISMED during the study period, 135 (96%) provided informed consent and were included in the study. From those, 78% (105/135) and 22% (30/135) were men and women, respectively, from one to 87 years old (≤10: 6%; [11–20]: 13%; [21–40]: 47%; [41–60]: 21%; >60: 13%). Eighty (59%) patients were leishmaniasis-confirmed cases. Excluding the patients with CM contamination (n = 5) we estimated that only 60% of the cases (45/75; 33 CL and 12 MCL cases) would have been diagnosed by PDE alone, and 90.6% (68/75; 51 CL and 17 MCL cases) if only CM was conducted. Thirty-eight out of 75 cases (50.6%) were positive for both PDE and CM (28 CL and 10 MCL cases), showing a moderate agreement between both diagnostic techniques (K = 0.43; 95% CI: 0.29–0.58).

The prevalence (71.3% CL and 28.7% MCL) and demographic characteristics of leishmaniasis confirmed cases are shown in Table 1. Among the MCL cases, 19 presented a scar from an old cutaneous lesion and 4 presented active concomitant cutaneous and mucosal lesions. The majority of leishmaniasis cases were found in men (83.8%), but taking into account the relationship between the number of confirmed and suspected cases for each sex, no statistical difference was observed (p = 0.4) (men: 67 confirmed cases/105 suspected cases (p = 0.6): women: 13 confirmed cases/30 suspected cases (p = 0.4)). The age range most affected was between 21–40 years old (58.8%; p = 0.004) with a population median age being 32.5 years old. All patients ≤ 20 years old presented a CL form. Forty-three cases (53.8%) were primo-infections, and two MCL cases without history of cutaneous lesions were classified as primary lesions (2.4%) (Table 1).

**Table 1:** Patient characteristics by clinical manifestation of leishmaniasis disease attending the IIBISMED Service in Cochabamba Department between 2014 and 2015.

	CL	MCL	TOTAL
	No. (%) patients	No. (%) patients	No. (%) patients
**Patients**	57 (71.3)	23 (28.7)	80 (100)
Sex*			
Male	46 (68.7)	21 (31.3)	67 (83.8)
Female	11 (84.6)	2 (15.4)	13 (16.3)
Age*			
Median (SD)			32.5 (16.6)
≤10	6 (100)	0 (0)	6 (7.5)
11–20	8 (100)	0 (0)	8 (10)
21–40	33 (70.2)	14 (29.8)	47 (58.8)
41–60	9 (64.3)	5 (35.7)	14 (17.5)
>60	1 (20)	4 (80)	5 (6.3)
**Episode**			
Primo-infection	43 (95.6)	0 (0)	43 (53.8)
Secondary-infection	14 (40)	21 (60)	35 (43.8)
Other**	0 (0)	2 (4.4)	2 (2.4)

* Association was found between the clinical form and the age range (p = 0.004).

** Primary lesions: MCL patients with no history of CL or visible scars.

The location, number, and type of lesions, as well as time of evolution are shown in Table 2. Among CL cases, infected ulcerative lesions were the most common (57.9%). Single lesions were the most frequent (61.4%) (Median: 1; Maximum number of lesions: 8), predominantly in lower members (43.9%) and with less than 6 months of evolution (82.5%). MCL patients presented mainly mucosal inflammation.

**Table 2:** Characteristics of clinical forms of leishmaniasis disease in patients attending the IIBISMED Service in Cochabamba Department between 2014 and 2015.

	CL	MCL
	No. (%) patients	No. (%) patients
**Cases**	57 (71.3)	23 (28.7)
**Type of cutaneous lesion**		
Ulcerative infected	33 (57.9)	2** (50)
Ulcerative non infected	19 (33.3)	1** (25)
Other clinical forms*	5 (8.8)	1** (25)
**Number of cutaneous lesions**		
1	35 (61.4)	2** (50)
>1	22 (38.6)	2** (50)
**Location (cutaneous)**		
Upper member	12 (21.1)	1** (25)
Lower member	25 (43.9)	0 (0)
Face	13 (22.8)	1** (25)
Neck	1 (1.8)	0 (0)
Trunk	1 (1.8)	0 (0)
More than one location	5 (8.8)	2** (50)
**Location (mucosal)**		
Nostrils		8 (34.8)
Mouth		3 (13)
Larynx		4 (17.4)
More than one location		8 (34.8)
**Time of evolution**		
≤ 6 months	47 (82.5)	7 (30.4)
6 months—1 year	5 (8.8)	3 (13)
> 1 year	5 (8.8)	13 (56.5)

CL: Cutaneous leishmaniasis; MCL: Mucocutaneous leishmaniasis.

* Nodular, infiltrative, proliferative and verrucous lesions.

** Patients with MCL that presented concomitant active cutaneous lesions.

Information about the previous treatment in patients presenting a second episode (same lesion or other lesions in different locations) is summarized in Table 3. Fourteen patients were considered as treatment failure (considering those who had received a complete treatment): including ten patients with CL and four patients with MCL. Glucantime (meglumine antimoniate) was the most frequently used drug (85%), followed by amphotericine B (5%). No information on the treatment and its duration was obtained for the rest of patients (10%). The large majority of patients receiving a second treatment completed it in the past (14/20; 70%) and, from those, 42.9% (n = 6) showed complete recovery for the past leishmaniasis episode. One primo-infection patient died during the treatment after an apparent recovery, however the cause of death was not recorded, although he was reported to have severe malnutrition.

**Table 3:** Information about the previous treatment in the 35 patients presenting a second episode of the disease.

	CL	MCL	TOTAL
	No. (%) patients	No. (%) patients	No. (%) patients
**Treatment Before the Current Episode (No = 35)**			
Previous treatment same lesion	11 (78.6)	2 (9.5)	13 (37.1)
Previous treatment other lesions	2 (14.3)	5 (23.8)	7 (20)
Not treated	1 (7.1)	14 (66.7)	15 (42.9)
**If treated, drug used (No = 20)**			
Glucantime	13 (100)	4 (57.1)	17 (85)
Amphotericine B	0 (0)	1 (14.3)	1 (5)
No information	0 (0)	2 (28.6)	2 (10)
**If treated, dosage (No = 20)**			
Complete	10 (76.9)	4 (57.1)	14 (70)
Not complete	1 (7.7)	2 (28.6)	3 (15)
No information	2 (15.4)	1 (14.3)	3 (15)
**If complete treatment for the same lesion, resolution (No = 14)**			
Complete Recovery	4 (40)	2 (50)	6 (42.9)
Partial recovery	5 (50)	0 (0)	5 (35.7)
No recovery	1 (10)	0 (0)	1 (7.1)
No information	0 (0)	2 (50)	2 (14.3)

CL: Cutaneous leishmaniasis; MCL: Mucocutaneous leishmaniasis.

### Cytokine profiles differ between CL and MCL clinical forms

**Fig 1:**
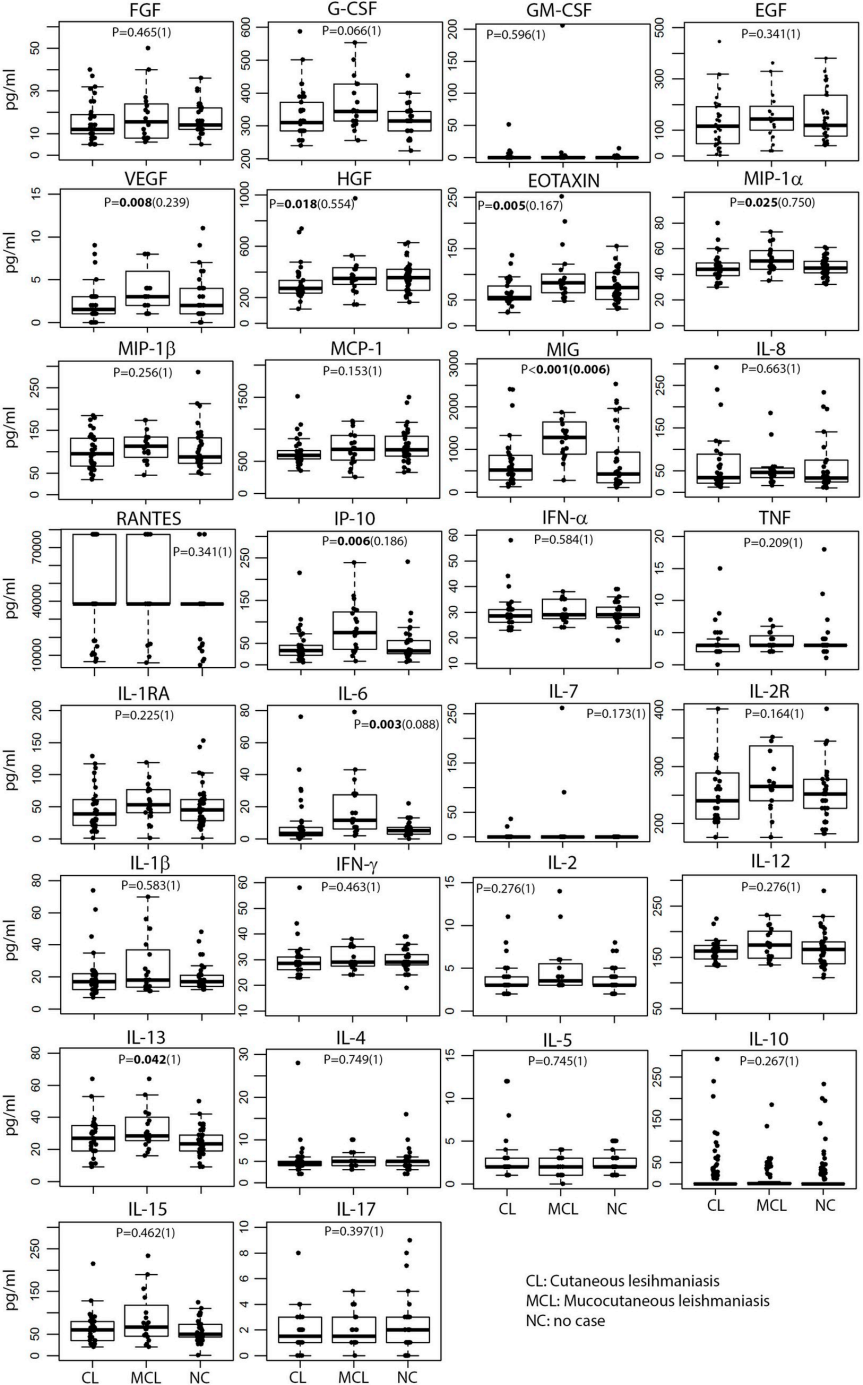
Differences in cytokine concentrations between CL, MCL and no-cases. Box plots representing the median and interquartile range of each marker concentration (pg/mL). Levels between both groups were compared by the Kruskal-Wallis Chi-squared test adjusting for multiple comparisons by using the Bonferroni correction (P-values in brackets). Significant P-values (<0.05) are in bold.

Higher concentrations of several cytokines were found in the MCL clinical cases compared to CL cases (Fig 1): the growth factors VEGF (P = 0.008) and HGF (P = 0.018); the chemokines eotaxin (P = 0.005), MIP-1α (P = 0.025), MIG (P<0.001) and IP-10 (P = 0.006); the pro-inflammatory cytokine IL-6 (P = 0.003) and the T_H_2 cytokine IL-13 (P = 0.042). However, after correcting for multiple comparisons only MIG remained significantly higher (P = 0.006). Accordingly, higher concentrations of most of these cytokines were associated with the MCL phenotype in the univariable regression models, however, after the stepwise multiple regression selection and adjusting by age, only eotaxin and MIG remained significant (Table 4).

**Table 4:** Univariate and multivariable linear regression models to assess the effect of the MCL clinical manifestation on cytokine concentrations. Linear regression models to assess the effect of MCL on cytokines concentrations.

	Coef. (p-value)		
	Univariate	Multivariate*	Final model**
MIP-1α	3.34 (**0.029**)		
G-CSF	2.54 (0.075)		
HGF	1.34 (0.064)		
VEGF	0.79 (**0.048**)		
Eotaxin	2.72 (**0.004**)	2.91 (**0.008**)	2.37 (**0.04**)
IP-10	1.18 (**0.006**)		
MIG	1.30 (**0.002**)	1.28 (**0.005**)	1.21 (**0.007**)
IL-6	0.73 (**0.012**)		
Age	0.058 (**0.010**)		0.03 (0.237)
Sex	0.847 (0.323)		

* Those cytokines, chemokines or growth factors significantly higher in MCL in the univariable models were included in the stepwise backward multiple regression selection, and the cytokines selected were included in the final multivariable model adjusted by age. Significant p-values (<0.05) are in bold.

** Final multivariate model adjusted by age.

### Cytokine profiles do not differ between primo and secondary infections

None of the cytokines presented different concentrations between primo-infections and secondary infections due to treatment failure. Only the growth factor EGF showed a higher trend in the secondary infections, but the difference was borderline (P = 0.055), and not statistically significant after multiple comparisons (P = 1) (S2 Fig).

## Discussion

In South America, parasitological diagnosis remains the reference standard in diagnosis of CL because of its high specificity, whereas the sensitivity varies over a wide range, and is much lower in MCL cases due to the scarcity of amastigotes [17]. In this study, nearly 60% of the 135 CL and MCL suspects tested by microscopy and culture were confirmed to be leishmaniasis cases. This result is consistent with the prevalence previously found in the tropic of Cochabamba, where 64% of cutaneous lesions proved to be leishmaniasis, with the rest being from other etiological origins [18]. However, it must be pointed out that some cases are missed due to the low sensitivity of the two diagnostic methods [17]. Until more accurate tools are implemented (e.g. PCR) all patients should be screened by both tests to minimize the risk of false negatives. As found in the current study, by only using PDE which is a more subjective technique, 40% of the cases would have been missed due to its low sensitivity that ranges from 15 to 70% [19]. On the contrary, by using CM alone, only 9.4% of cases would have been overlooked. Five patients could be diagnosed solely by PDE due to culture contamination (6.3%), which were mainly MCL cases. In our study, the rate of culture contamination was low (9/135; 6.7%), and stemmed only from fungal microorganisms. The low frequency of contamination was most likely due to the high concentration of antibiotic in the culture medium, the proper cleaning of the lesions before sampling and the expertise of the laboratory staff [10]. The over-infection of lesions was frequently due to lack of hygiene and/or the use of domestic treatments, causing a decrease in the sensitivity of the diagnostic techniques if not solved [20].

In this study, 18% of cases were considered to be treatment failures according to the criteria described above [1,7,12,13]. Llanos-Cuentas and colleagues found that the risk factors associated with treatment failure were young age, presence of an additional lesion and infection with *L*. *braziliensis* [12]. Patients not included in the main groups (primo-infection or treatment failure) had different characteristics and were difficult to identify. The majority did not finish treatment, and others used different plants or solvents to dry the wound or simply burn it. Occasionally, the lesion self-resolved leaving a scar, but this is not a guarantee of parasitological cure, and MCL could still appear after several years [1,20]. Mucosal lesions required hospitalization to be treated and may be fatal in some cases [1].

Since the study only included patients arriving passively to be diagnosed, a considerable number of undiagnosed might have been missed [1,4,21]. Access barriers to case management were associated with the absence of diagnostic methods and trained staff in rural areas, their cost, limited accessibility to health centres (scarce and expensive transport, poor road conditions, long travel distances), and adverse climate conditions [4,10,20,21,22]. The local languages (Quechua and Aymara) and the stigmatization of the disease also hampers communication with the patients [4,22].

Locations where patients may have acquired the infection were deduced from interviews, but in some cases this information was not available. As found in other studies, most infections appeared to be acquired in the tropical region of Chapare province (56%), in the department of Cochabamba [22,23]. The risk of transmission in this area is the highest in the region, as shown by entomological studies [24,25,26]. The traditional sylvatic pattern of transmission is the most prevalent in the area [3,22,23,25], but migrations to endemic areas and leishmaniasis urbanization are also potential means of infection as seen in other countries [22,27,28]. A large percentage of our population with therapeutic failure had a history of visiting endemic areas for only short periods, therefore lacking previous exposure to parasite antigens and sand fly saliva that confer protective immunity [5].

In the present study, the age range most affected was between 21–40 years old and infections probably occurred during work activities, with more frequent lesions found in the exposed areas of the body, mainly lower members [1,22,28,29]. These patients described field activities as farming, fruit and vegetable agriculture, or coca cultivation. Occasionally, women and children accompanied men to their job activities [23,29]. However, it is worth noting that the number of women self-presenting was three times lower (30/135) than men. This may be related to a genuine lower prevalence among women and/or to differences in access to health services associated to sex as described before [28,29,30]. In addition, leishmaniasis shows sex-related differences regarding physiology, immune responses, drug metabolism and progression of the disease [31].

Cellular immunity is essential for controlling intracellular pathogens like *Leishmania*, and of primary importance in the outcome of this infection. In the mouse model of *L*. *major*, resistance and susceptibility are determined by functionally distinct T cell subsets [32,33], with a predominant T helper (Th)1 response leading to the control of the infection, and a Th2 response increasing susceptibility and disease progression [34,35]. However, an exaggerated Th1 response has been associated with tissue damage [36,37]. Our results showing higher concentrations of cytokines in MCL agree with previous studies reporting that lymphocyte activation, proliferation and cytokine secretion upon stimulation with *Leishmania* antigens are higher in MCL compared with CL [37,38], probably related to the severity of tissue destruction in MCL. Among the analytes increased in MCL, we found mainly growth factors (VEGF and HGF) and chemokines (eotaxin, IP-10, MIG and MIP-1α), as well as the pro-inflammatory cytokine IL-6 and the Th2 cytokine IL-13. Higher concentrations of growth factors in MCL may be related to the more severe and extensive tissue destruction and the need of a more active tissue repair. VEGF is associated with changes in the vasculature during wound healing, and has been suggested to be important in the development and resolution of leishmanial lesions [39]. Similarly, HGF is a potent angiogenic factor having a major role in wound healing, acting upon epithelial and endothelial cells, and on haemopoietic progenitor cells and T cells [40].

The higher concentration of several chemokines in MCL could also be related to the more extensive wound healing, as they stimulate the migration of multiple cell types to the wound site, particularly inflammatory cells [41], and contribute to the regulation of re-epithelialization, tissue remodelling, and angiogenesis [42]. In addition, chemokines may have antimicrobial activity involved in parasite clearance. Eotaxin may chemoattract eosinophils to the infection site for *Leishmania* clearance [43]. Similarly, IP-10, MIG and MIP-1α, three Th1-mobilizing chemokines produced by leishmaniasis lesion cells, recruit monocytes, macrophages, and activated T cells to sites of infection, contributing to wound healing and parasite elimination [44,45]. Neutrophil derived MIP-1α has also been shown to be essential for the recruitment of dendritic cells to the infection site that will further direct the development of an adaptive immune response [46].

The higher concentration of IL-6 in MCL agrees with a previous association with severity in visceral leishmaniasis [47,48]. This detrimental effect of IL-6 has been related to its suppressive effect on TNF production and Th1 responses, and its induction of Th2 responses, which is suggested to down-regulate macrophage microbicidal activity, facilitating *Leishmania* proliferation [47]. Finally, the higher concentration of IL-13 in MCL may have a dual role (mainly Th2 but also Th1 responses) that is influenced by parasite species and the host’s genetic background [48,49].

Among all these biomarkers, only eotaxin and MIG showed significantly higher concentrations in MCL versus CL after adjustment, indicating their relevant role in the immune response against the MCL clinical form. Their contribution in recruiting cells to the infected tissues may be favoring macrophage activation and parasite clearance; however, excessive production of these molecules could potentially lead to uncontrolled inflammation and tissue destruction [45,50]. Thus, it is difficult to disentangle whether the higher concentrations of these chemokines in MCL versus CL are a positive response to a more extensive infection, or are in fact contributing to the more generalized inflammation and tissue damage observed in MCL cases. The absence of association between the levels of cytokines and primo-infections or secondary infections suggests similar cellular immune responses among them independently of the type of infection.

One limitation of the study is that the analyses did not consider the *Leishmania* species causing the CL and MCL lesions as those were not identified. However, based on previous studies in the same area, we would expect that over 85% of the cases were caused by *L*. *braziliensis* [2,30]. The intrinsic variability of the circulating *Leishmania* species influences treatment efficacy, thus it is important to be considered for the control of the disease [2]. In addition, the related lack of data on resistance to antimonials is also important for disease control, but its incorporation into the diagnostic routine is not realistic in the Bolivian context. The study does not include healthy individuals as control group for the comparison of cytokine concentrations with leishmanial cases. We only had sera from patients with lesions compatible with leishmaniasis but not confirmed as cases, however some cases may have been missed due to the low sensitivity of the current diagnostic methods [17]. Finally, quality and quantity of inflammatory cells in the lesions may differ between clinical phenotypes and patients, affecting the local cytokine profiles, and this may not be reflected in the serum cytokine profiles. Future studies should address the local cellular immune response in the lesions to better understand its association to wound healing and evolution of treatment.

In conclusion, the results of the present study provide additional information on the epidemiological, clinical and immunological situation of TL in Bolivia. Although parasitological diagnosis remains the reference standard for leishmaniasis the diagnostic techniques evaluated in the present study evidence that more accurate tools must be implemented in the future to avoid false negatives. Also, further studies must be done in a larger number of patients relating clinical manifestations with species characterization considering that *Leishmania* species identification might be helpful for better disease management. Regarding the immunological analysis these results also highlight that further research would certainly present a further step towards improving knowledge of potential biomarkers of susceptibility and disease progression.

## Supporting information

S1 Fig Semi-structured questionnaire. Demographic, epidemiological and clinical data collected from the patients recruited for the study.
(TIF)

S2 Fig Differences in cytokine concentrations between primo and secondary infections. Box plots representing the median and interquartile range of each marker concentration (pg/mL). Levels between both groups were compared by the Kruskal-Wallis Chi-squared test adjusting for multiple comparisons by using the Bonferroni correction (P-values in brackets). Significant P-values (<0.05) are in bold.
(TIF)
