# Supplementary figures and images for: Clinical and immunological characteristics of tegumentary leishmaniasis cases in Bolivia
Source: PLoS Negl Trop Dis. 2021 Mar 5;15(3):e0009223. doi: 10.1371/journal.pntd.0009223 (PMC7968743; doi:10.1371/journal.pntd.0009223)

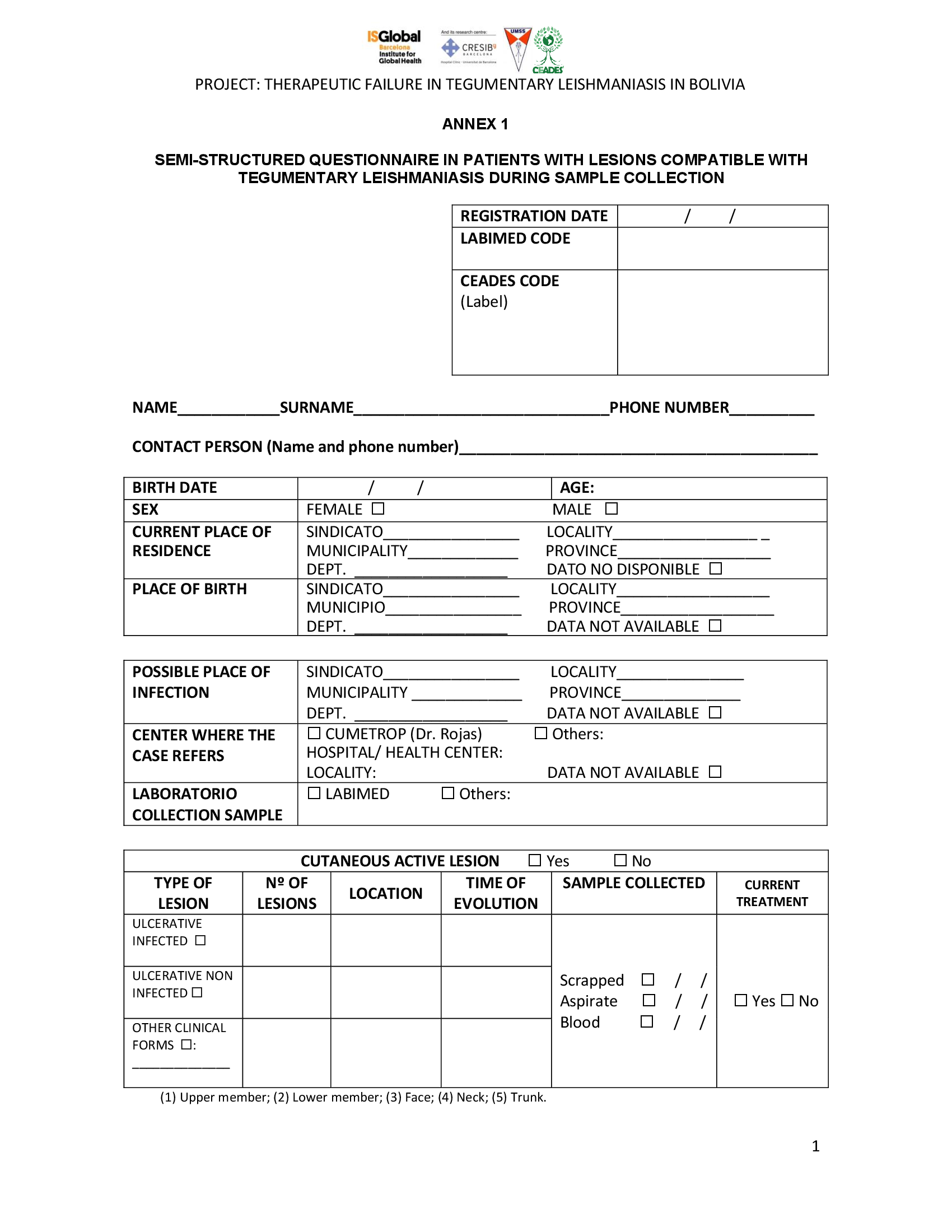

Supplement: S1 Fig — Demographic, epidemiological and clinical data collected from the patients recruited for the study. (TIF) [file pntd.0009223.s001.tif]

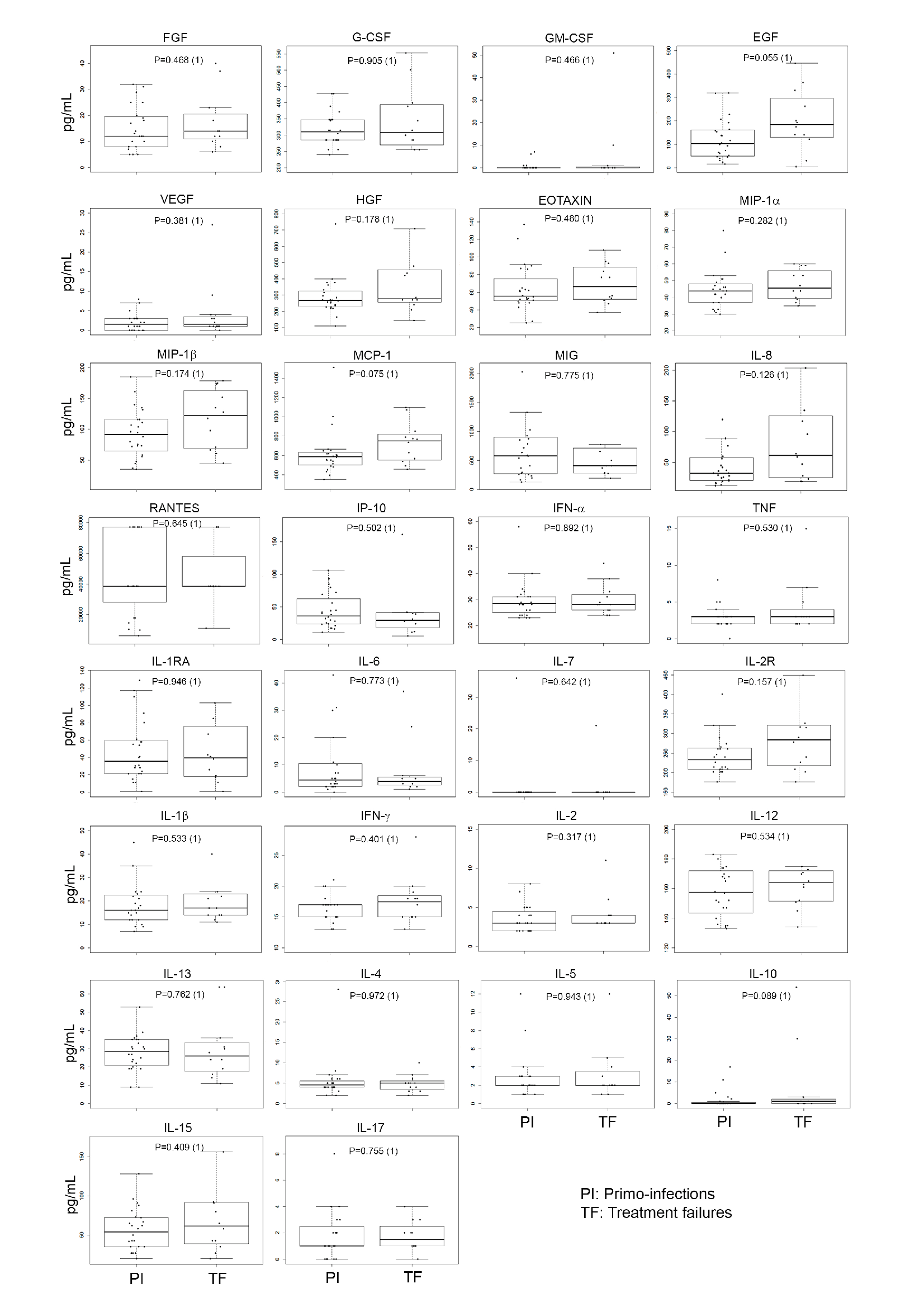

Supplement: S2 Fig — Box plots representing the median and interquartile range of each marker concentration (pg/mL). Levels between both groups were compared by the Kruskal-Wallis Chi-squared test adjusting for multiple comparisons by using the Bonferroni correction (P-values in brackets). Significant P-values (<0.05) are in bold. (TIF) [file pntd.0009223.s002.tif]
